# Multicenter reliability study of the universal long bone nonunion classification

**DOI:** 10.1016/j.jcot.2025.103302

**Published:** 2025-12-08

**Authors:** Anton A. Semenistyy, Leonid N. Solomin, Artem V. Komarov, Roman Y. Mitsikov, Borislav G. Tasev, Andrey N. Mironov

**Affiliations:** a Department of Orthopedics and Traumatology, Faculty of Medicine, Medical University of Sofia, Blvd. Akad Ivan Geshov 15, Sofia, 1431, Bulgaria; b Department of Orthopedic Surgery, Vreden National Research Orthopedic Centre, Akad. Baykov str. 8, Saint Petersburg, 195427, Russian Federation; c Department of Military Traumatology and Orthopedics, S. M. Kirov Military Medical Academy, Akad. Lebedev str. G, Saint Petersburg, 194044, Russian Federation; d Department of Traumatology and Orthopedics, Rostov State Medical University, Suvorov str. 119, Rostov-on-Don, 344022, Russian Federation; e Department of Traumatology, “N.I. Pirogov” University Emergency Hospital, Blvd. Gen. Totleben 21, Sofia, 1606, Bulgaria; f Department of Traumatology and Orthopedics, Moscow, “Acad. G.M. Savelieva” City Clinical Hospital, Lobachevskogo str.42/1, Moscow, 119415, Russian Federation

**Keywords:** Classification, Validation, Nonunion, Delayed union, Pseudoarthrosis, Hypertrophic, Normotrophic, Oligotrophic

## Abstract

**Background:**

A universal classification providing a clinically relevant and anatomically comprehensive framework for long bone nonunions has been recently introduced. This study aimed to evaluate its inter- and intra-observer reliability and compare its performance with the widely used Weber–Cech classification.

**Methods:**

This multicenter, three-stage validation study included 191 cases meeting the FDA definition of nonunion. Four expert raters participated. In Stage 1, cases were classified using existing systems: AO/OTA for anatomical location and Weber–Cech for biological type. In Stage 2, 133 eligible cases were independently classified using the Universal Long Bone Nonunion Classification (ULBNC) in two rounds, two weeks apart. Stage 3 involved refinement of classification criteria based on feedback and statistical analysis, followed by re-assessment of 90 cases. Inter-observer reliability was assessed using free-marginal Fleiss' kappa; intra-observer reliability using Cohen's kappa with linear weighting.

**Results:**

Substantial to almost perfect inter-observer agreement was observed for type classification (κ = 0.85), with the highest reliability in diaphyseal nonunions (κ = 0.90). Incorporating pathological mobility significantly improved agreement compared to Weber–Cech (κ = 0.38, p < 0.05). Periarticular nonunions showed substantial agreement (κ = 0.72). Group-level agreement improved from moderate (κ = 0.42–0.57) to substantial (κ = 0.79–0.82) after refining criteria. Subgroup agreement was excellent (κ = 0.89–1.00). Intra-observer reliability ranged from substantial to almost perfect across all levels.

**Conclusion:**

ULBNC is a reliable and reproducible classification system for long bone nonunions. Incorporation of clinical features—such as pathological mobility, alignment, and correction strategy—enhances its clinical utility and supports standardization in treatment and research.

## 1 Introduction

Nonunion is one of the most challenging complications in orthopaedic trauma, occurring in approximately 1.9–10 % of long bone fractures, depending on fracture location, biological conditions, patient risk factors, and surgical technique.^1^ Despite advances in fixation methods and bone-healing technologies, both the diagnosis and treatment of nonunion remain inconsistent, largely due to the lack of a universally accepted and clinically meaningful classification system.^2^^,^^3^

The most widely used system remains the radiographic classification by Weber and Cech (1976), which categorizes nonunions as hypertrophic, normotrophic, oligotrophic, or atrophic based on appearance.^4^ However, its core assumption—that radiographic morphology reflects vascularity—has been questioned.^5^

Ilizarov shifted attention to pathological mobility, distinguishing stiff (PM < 7°) from lax nonunions.^6^ Paley expanded this by adding a deformity-based subclassification of stiff types.^7^ Chi-Chuan incorporated fixation stability as a criterion.^8^ Calori's Nonunion Scoring System (NUSS) quantifies severity using clinical and patient factors but lacks anatomical or morphological structure and offers limited treatment guidance.^9^, ^10^, ^11^

Solomin proposed the Universal Long Bone Nonunion Classification (ULBNC), using the AO localization code to differentiate nonunions anatomically and ensure continuity between fracture and nonunion classification.^2^^,^^12^ ULBNC classifies nonunions by localization, biological type (based on radiography and pathological mobility), fixation type and stability, and the presence and method of deformity correction.

The aim of this study was to assess the inter- and intra-observer reliability of the ULBNC.

## 2 Methods

### 2.1 Study design

This was a prospective, multicentre, three-stage reliability study conducted in accordance with established guidelines for validating orthopaedic classification systems.^13^ All cases were anonymized and independently assessed by expert raters blinded to each other's evaluations.

### 2.2 Inclusion and exclusion criteria

Patients were eligible if they had a radiographically confirmed long bone nonunion, defined by the United States Food and Drug Administration (FDA) as a fracture unhealed ≥9 months after injury with no radiographic progression for ≥3 months.^14^ Septic and atrophic nonunions were excluded because their management requires resection of necrotic or infected bone, creating a segmental defect that must be classified separately.^15^

Raters could refrain from classifying a case if they judged it to represent a segmental defect or if the available imaging or clinical data were insufficient. Any case excluded by at least one rater was omitted from analysis for all raters.

### 2.3 Requirements for clinical cases

For **stage 1** each case included the following mandatory dataset:

1. **Imaging:** standard anteroposterior and lateral radiographs
2. **Clinical examination:** swelling, pain at rest, and pain during weightbearing

For **Stages 2 and 3** mandatory data included:

1. **Imaging:** standard anteroposterior and lateral radiographs. Computed tomography (CT) for all periarticular nonunions with multiplanar (coronal, sagittal, axial) and 3D reconstructions. Deformity analysis covered angular and torsional deformities (degrees), translation (mm), and shortening (mm).
2. **Clinical examination:** swelling, pain at rest, and pain during weightbearing and assessment of **pathological mobility (PM)** (presence and degree of abnormal motion at the nonunion site), recorded both preoperatively and intraoperatively.

### 2.4 Pathological mobility assessment

PM was assessed clinically and intra-operatively under fluoroscopic control. Clinical examination served only as a screening tool, as it detects only gross instability and is unreliable in two-bone segments (forearm, lower leg), where the adjacent bone restricts motion, and in periarticular locations where joint movement may mimic pathological mobility.

Accurate assessment was performed intra-operatively after hardware removal and, for tibial nonunions, fibular osteotomy. Under fluoroscopy, the nonunion site was stressed in the plane of deformity in both directions to obtain two maximal, opposite deviations from the neutral axis. Pathological mobility was quantified as the angular difference (degrees) between these positions.

In the forearm, osteotomy of the second bone was not required; mobility was evaluated by direct manipulation of the fracture fragments and measured using the same fluoroscopic or photographic technique.

### 2.5 Raters

Four senior orthopedic trauma surgeons participated, each with at least ten years of experience and a minimum annual caseload of 10 fracture nonunions over the previous three years. Аll observers received standardized training and reference materials for the classification systems.

### 2.6 Phases of evaluation

A total of 191 long bone nonunion cases were evaluated over a three-stage classification process. This resulted in a total of 2188 individual assessments across all types, groups, and subgroups of nonunions.

#### 2.6.1 Stage 1 – evaluation of precursor classification systems to ULBNC

Classification of 191 cases (764 assessments) using conventional systems:

• AO Fracture Classification – anatomical localization and articular involvement (extra-articular, partial articular, complete articular).^12^
• Weber–Cech classification – biological type of nonunion.^4^

The aim of this stage was to identify the limitations of existing classification systems and to determine case eligibility for further analysis.

#### 2.6.2 Stage 2 – primary validation of ULBNC

A subset of 133 cases (after exclusion of 58 cases following Stage 1) was independently classified using the ULBNC by four expert raters. Classifications were repeated after a two-week washout period to assess intra-observer reliability, yielding 1064 total assessments. Anatomical localization was predefined by the authors and is presented in Table 1.

**Table 1:** The anatomical distribution of classified cases.

Localization	N of cases	% of cases
Diaphyseal	**97**	**72.9**
12	13	9.8
2R2	8	6
2U2	10	7.5
32	34	25.6
4T2	24	18.0
4F2	8	6
Periarticular	**36**	**27.1**
11	4	3
13	0	0
2U1	0	0
2R1	0	0
2U3	0	0
2R3	2	1.5
31	5	3.8
33	7	5.3
4T1	1	0.7
4F1	0	0
4T3	10	7.5
4F3	7	5.3
Overall	**133**	**100 %**

Inter- and intra-rater reliability were evaluated separately for diaphyseal and periarticular nonunion types, as well as for their respective groups and subgroups. Inter-rater reliability was analyzed independently for each major anatomical and morphological category: (1) diaphyseal Type A, (2) diaphyseal Types B and C, and (3) periarticular nonunions, reflecting differences in classification structure.

For group-level analysis, cases in which a rater assigned different types (Type A vs. Type B/C) between rounds were excluded, as variation in type allocation invalidates group comparison. Consequently, group-level reliability was assessed in 131, 129, 131, and 127 cases for raters 1–4, respectively.

Because subgroup structure is uniform across all diaphyseal nonunions but differs in periarticular cases, inter-observer reliability was assessed separately for diaphyseal and periarticular subgroups.

#### 2.6.3 Stage 3 – classification optimization

After analysis of Stage 2 results, the ULBNC criteria were refined to address areas of disagreement at the group level. A total of 90 cases were randomly selected for re-evaluation using the revised criteria, comprising 30 diaphyseal Type A, 30 diaphyseal Types B/C, and 30 periarticular nonunions. Each case was independently reviewed by four observers, yielding 360 assessments. The objective was to determine whether the refinements improved inter-observer agreement.

The new criteria included:

• definitions of acceptable displacement for each anatomical region;
• indications for acute versus gradual correction of hypertrophic nonunions;
• standardized criteria for assessing fixation stability.

To evaluate their impact, inter-observer agreement from Stage 3 was compared with cumulative data from Stage 2 (both rounds), analyzed separately for diaphyseal Type A, diaphyseal Types B/C, and periarticular nonunions.

### 2.7 Universal long bone nonunion classification

#### 2.7.1 Localization

**Fig. 1:**
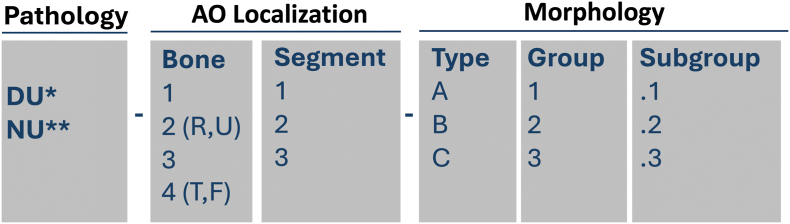
The alphanumeric code used for nonunion classification. “Ankle” segment (44) is not used. The localization 4T3 is used for medial or posterior malleolus. The localizations 4F2 and 4F3 are used for lateral malleolus. ∗ DU – delayed inion, 1-2ECT, ∗∗ NU – nonunion, >2ECT

The anatomical localization of the nonunion follows the AO/OTA fracture classification^9^ principles, distinguishing between bones (1, 2R, 2U, 3, 4T, 4F) and segments (1,2,3) allowing for precise topographical description (Fig. 1).

1. Diaphyseal segment

Types.

**Fig. 2:**
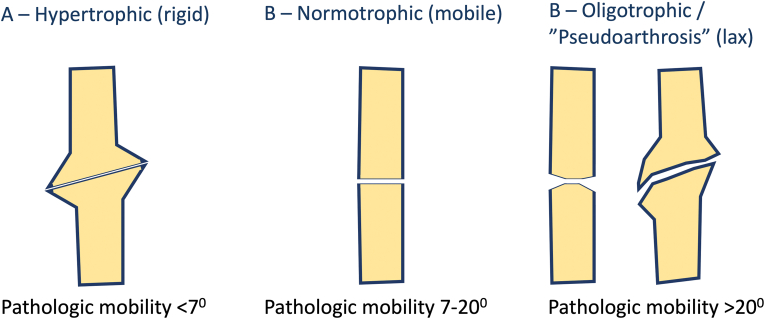
Types of diaphyseal nonunions.

Pathological mobility (PM) was adopted as the primary criterion for differentiating nonunion types. The classification of diaphyseal nonunions by type is illustrated in Fig. 2. In the presence of an internal fixation device, the absence of PM suggests stable fixation, whereas its presence indicates instability. In such cases, definitive classification of the nonunion type may only be possible intraoperatively following removal of the hardware. Specifically for the tibia (4T2), pathological mobility should be assessed after both implant removal and fibular osteotomy.

Groups.

**Fig. 3:**
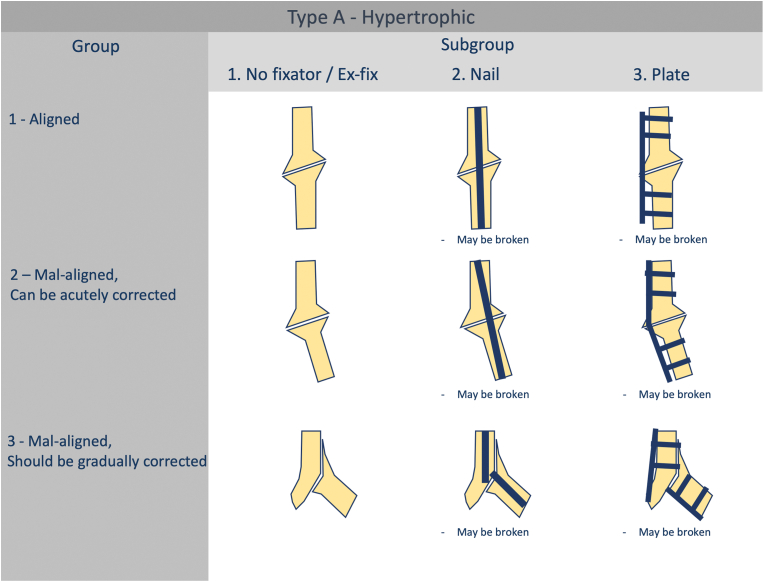
Groups and subgroups of diaphyseal hypertrophic (Type A) nonunions.

**Fig. 4:**
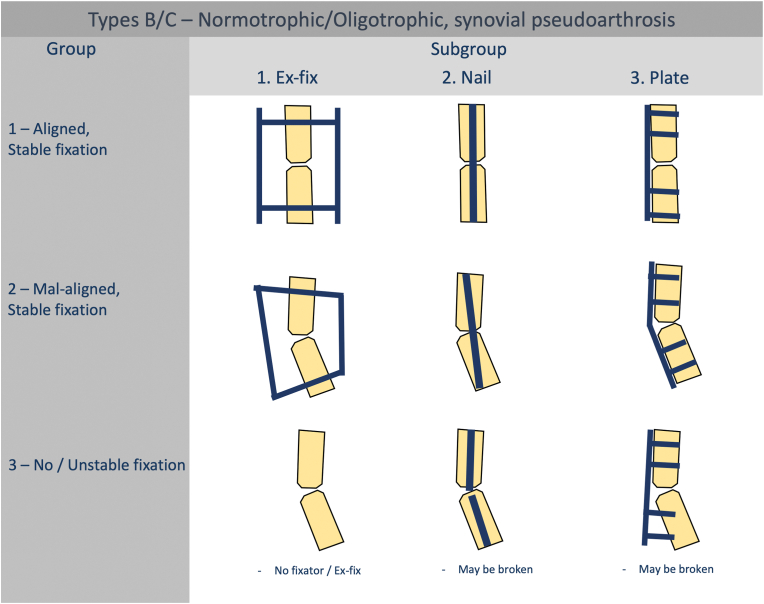
Groups and subgroups of diaphyseal normotrophic/oligotrophic (Types B and C) nonunions.

For diaphyseal nonunions, the classification distinguishes between Type A and Types B/C based on different criteria. In Type A nonunions, the primary determinant is alignment (Fig. 3). In contrast, Types B and C depend mainly on hardware stability and the quality of reduction (Fig. 4).

The recommended criteria for group determination, developed after Stage 2 validation, are summarized in Table 2. Assessment of acceptable alignment followed established normative reference lines and angles described by Paley.^16^

**Table 2:** The recommended criteria for group determination in diaphyseal nonunions.

Type and group	Localization				
	**12 - humerus**	**22 - forearm**	**32 - femur**	**4T2 - tibia**	**4F2 - fibula**
A1. Aligned	Angulation **≤20^0^** Malrotation **≤20^0^** Shortening **≤30 mm**	No deformity (any deformity = mal-alingment)	Accepted angulation and translation – all lines and angles within reference values Normal torsion – rotational profile within reference values Shortening **≤15 mm**		Any displacement with preserved ankle joint congruency and syndesmosis integrity
A2. Mal-aligned. Can be corrected acutely	Angulation **21-40^0^** Malroration **>20^0^** Any unaccepted translation∗	Angulation **≤20^0^** Shortening **≤10 mm** Any translation and malrotation	Any displacement (angulation, translation) resulting in shift of lines and angles out of reference values, required correction **≤20^0^** Malrotation - rotational profile outside reference values		Any displacement with ankle joint incongruency or syndesmosis rupture
A3. Mal-aligned. Should be corrected gradually	Angulation **>40^0^** Shortening **>30 mm**	Angulation **>20^0^** Shortening **>10 mm**	Angulation **>20^0^** Shortening **> 15 mm**		Ankle joint dislocation
B/C1 Stable hardware + acceptable reduction	No pathologic mobility + Angulation **≤20^0^** Malrotation **≤20^0^** Shortening **≤3 cm**	No pathologic mobility + No deformity (any deformity = mal-alignment)	No pathologic mobility + Accepted angulation and translation – All lines and angles are within reference values Normal torsion – rotational profile within reference values Shortening **≤15 mm**		Any displacement with preserved ankle joint congruency and syndesmosis integrity
B/C2 Stable hardware + unacceptable reduction	• No pathologic mobility + mal-alignment				
B/C3 Unstable/no hardware	• Any pathologic mobility = instability • Suspicion criteria for instability: • resorption around hardware • hardware migration • hardware breakage				

Subgroups.

Based on the presence and type of a fixator, nonunions are divided into subgroups as presented in Fig. 3, Fig. 4.

2. Periarticular segment

Types.

**Fig. 5:**
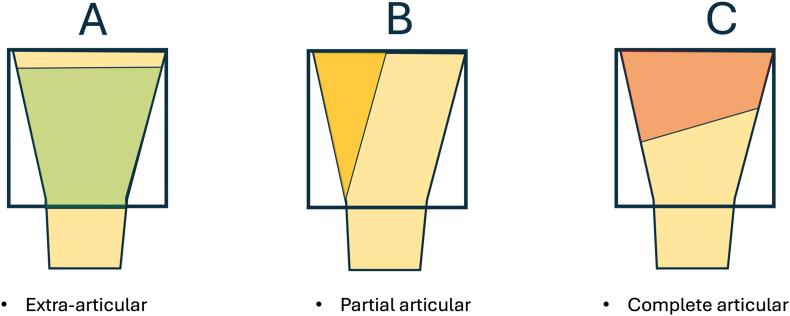
Types of periarticular nonunions.

**Fig. 6:**
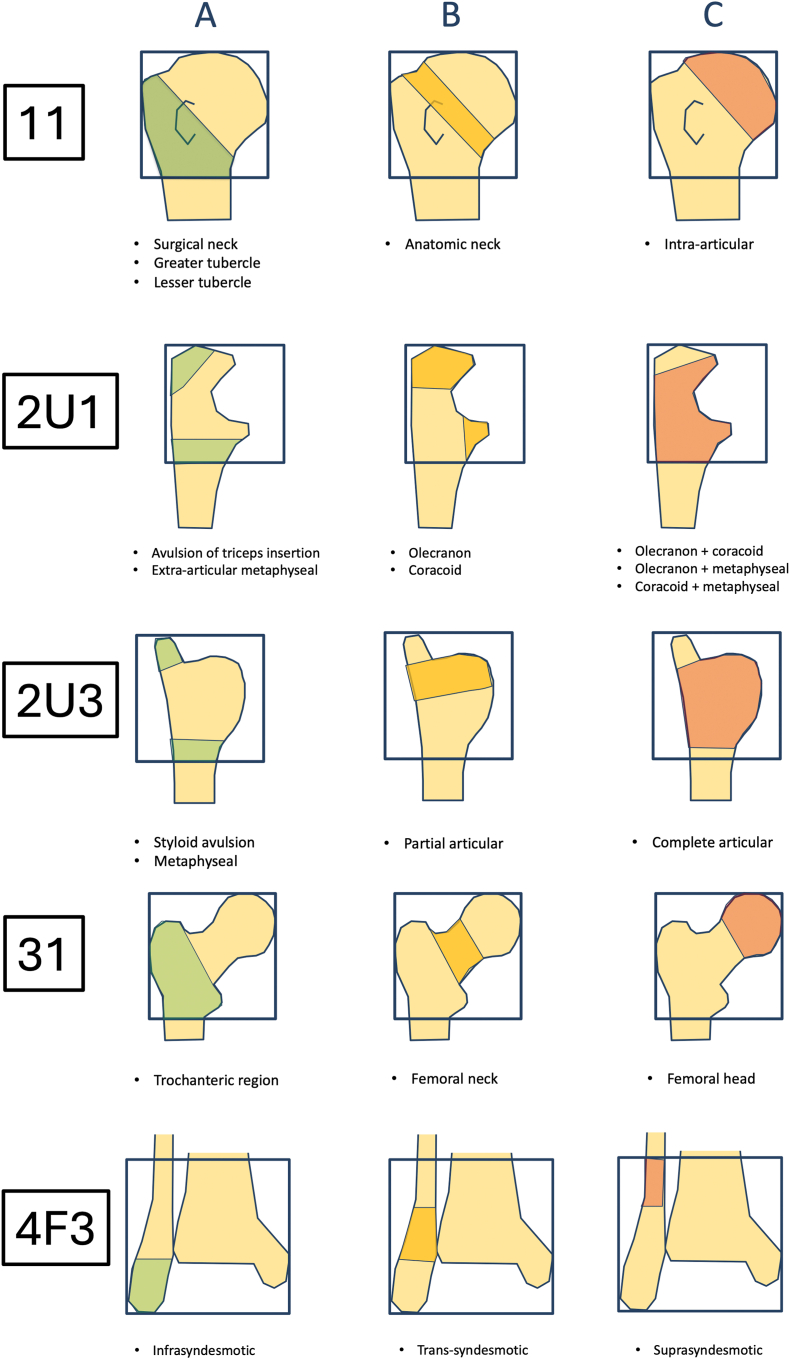
Types of periarticular nonunions for specific localizations: 11 – proximal humerus, 2U1 – proximal ulna, 2U3 – distal ulna, 31 – proximal femur, 4F3 – distal fibula.

The same principle as in AO/OTA fracture classification^12^ for articular region are used for distinguishing types of periarticular nonunions (Fig. 5). For specific periarticular regions (11, 2U1, 2U3, 31, 4F3) were added clarifications as presented below (Fig. 6)

Groups and subgroups.

**Fig. 7:**
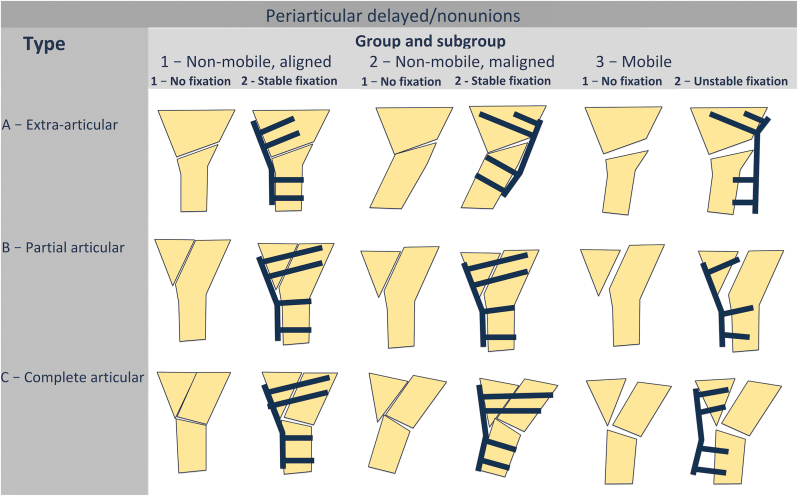
Groups and subgroups of periarticular nonunions.

The main criteria for group division in periarticular nonunions are pathological mobility and the quality of reduction. Because joint motion can obscure true instability, PM must be assessed under fluoroscopic control. If no clear movement is observed, the nonunion is considered non-mobile. Based on the presence and type of fixation, periarticular nonunions are further divided into subgroups (Fig. 7).

The recommended criteria for determining periarticular nonunion groups, developed after Stage 2 validation, are presented in Table 3, Table 4.

**Table 3:** Recommended criteria for determination the groups of periarticular nonunions. ∗Thresholds for acceptable intra-articular alignment (step off and inter-fragmentary diastasis) by anatomical localization are provided in Table 4.

Group	For all periarticular segments
1. Non-mobile (or stable hardware) + acceptable reduction	No evident pathological mobility during clinical examination and fluoroscopic investigation + • Extra-articular – angles within reference values • Intra-articular – congruent articular surfaces • Anatomical reduction of major fragments∗
2. Non-mobile (or stable hardware) + unacceptable reduction	No evident pathological mobility during clinical examination and fluoroscopic investigation + • Extra-articular – angles out of reference values • Intra-articular – incongruent articular surfaces • Non-anatomical reduction of major fragments∗
3. Mobile	• Any Pathologic mobility = instability **Criteria for instability:** • Resorption around the hardware • Hardware migration • Hardware breakage • Significant loss of joint function • Joint instability following avulsion fractures

**Table 4:** Recommended criteria for alignment evaluation in periarticular nonunions by anatomical localization.

**Localization**	**Unacceptable alignment criteria**	
**11**	• Angles out of reference values • Malrotation **> 20^0^** • Impingement	• Intra-articular step-off **> 2 mm** • Tuberosities displacement **>5 mm**
**13**	• Angles out of reference values • Malrotation **> 20^0^** • Epicondyle displacement **> 2 mm**	• Any articular incongruence • Impingement
**2R1**	• Intra-articular step-off, diastasis **> 2 mm** • Angles out of reference values	• Radiohumeral joint instability, subluxation, dislocation
**2U1**	• Angles out of reference values	• Intra-articular step-off, diastasis **> 1 mm**
**2R3**	• Angles out of reference values • Radial length – out of reference values	• Intra-articular step-off, diastasis **> 2 mm** • Radioulnar joint instability, subluxation, dislocation
**2U3**	• Angles out of reference values	• Intra-articular step-off, diastasis **> 2 mm**
**31**	• Angles, rotational profile out of reference values	• Intra-articular step-off, diastasis> **1 mm** • Trochanters displacement **>10 mm**
**33**	• Angles, rotational profile out of reference values	• Intra-articular step-off, diastasis **> 2 mm**
**4T1**	• Angles, rotational profile out of reference values	• Intra-articular step-off, diastasis **> 1 mm** • Tuberosity displacement **>2 mm**
**4F1**	• No consensus	
**4T3**	• Angles, rotational profile out of reference values	• Intra-articular step-off, diastasis **> 1 mm**
**4F3**	• Angles, rotational profile out of reference values • Fibular shortening **>2 mm**	• Intra-articular step-off, diastasis **> 1 mm** • Syndesmosis widening, incongruency

### 2.8 Statistical analysis

Inter-observer reliability was assessed using free-marginal Fleiss' kappa, appropriate for multi-rater categorical data without fixed marginal totals. Intra-observer reliability was calculated using Cohen's kappa with linear weighting. Kappa values were interpreted per Landis and Koch criteria: slight (0.00–0.20), fair (0.21–0.40), moderate (0.41–0.60), substantial (0.61–0.80), and almost perfect (>0.80).^17^ Pairwise comparisons of kappa values were performed using Z-tests based on standard errors derived from 95 % confidence intervals. To control for multiple comparisons, Bonferroni correction was applied, and the threshold for statistical significance was set accordingly. A p-value <0.05 was considered statistically significant unless adjusted by multiple testing correction. All analyses were performed using SPSS version 26 (IBM Corp., Armonk, NY, USA).

## 3 Results

### 3.1 Stage 1 – evaluation of precursor classification systems to ULBNC

Out of 191 cases, 24 were excluded by the raters for the following reasons: insufficient or low-quality radiological imaging in 14 cases; lack of adequate clinical data in 1 case; and 9 cases that did not meet the criteria for nonunion classification (e.g., atrophic nonunion or bone defects). 167 (87.4 %) cases were classified.

#### 3.1.1 Anatomical localization (AO/OTA fracture and dislocation classification)^12^

Out of 167 cases, the anatomical location was consistently identified by all four raters in 144 cases (86.2 %). Among these, 104 nonunions (72.2 %) were classified as diaphyseal and 40 (27.8 %) as periarticular. In 23 cases (13.8 %), disagreement was observed due to difficulties in classifying borderline anatomical zones: proximal humerus (zones 11/12) – 3 cases, proximal femur (31/32) – 4 cases, distal femur (32/33) – 6 cases, proximal tibia (4T1/4T2) – 5 cases, distal tibia (4T2/4T3) – 3 cases, and distal fibula (4F2/4F3) – 2 cases.

#### 3.1.2 Periarticular types (AO/OTA fracture and dislocation classification)^12^

A total of 40 cases were classified. Full agreement among all four raters was observed in only 20 cases (50 %). The overall inter-rater agreement was 65.83 %, with a Kappa of 0.49 (95 % CI: 0.31–0.66), indicating moderate agreement according to the Landis and Koch criteria. CT imaging was not provided at this stage, which may partially explain the limited agreement among raters.

#### 3.1.3 Biological type (Weber–Cech classification)^4^

Out of 104 diaphyseal nonunions, full agreement among all four raters was observed in only 39 cases (37.5 %). Among the 65 cases with disagreement, the majority involved difficulty in distinguishing between Type A and B/C - 38 (58.5 %) cases, between Type B and C – 21 (32.3 %) cases, and among all three types A, B, and C – 6 (9.2 %) cases. The overall inter-observer agreement was 58.65 %, (k = 0.38; 95 % CI: 0.28–0.48), indicating fair agreement.

### 3.2 Stage 2 – primary validation of ULBNC

#### 3.2.1 Inter-observer reliability

Types.

Inter-observer reliability across the entire classification system demonstrated substantial agreement (κ = 0.77) and increased to almost perfect agreement after the second round (κ = 0.85), although the difference was not statistically significant (p = 0.066). By type, diaphyseal nonunions consistently showed almost perfect agreement, with κ = 0.82 and 0.90 in rounds 1 and 2. Segment-specific analysis confirmed similarly high reliability for both femoral (κ = 0.94) and tibial (κ = 0.91) nonunions, with no significant difference between them (p = 0.480).

The addition of PM as a clinical criterion markedly improved classification reliability: κ increased from 0.38 with the Weber–Cech system to 0.82 and 0.90 (rounds 1 and 2) with the ULBNC (both p < 0.001).

For periarticular nonunions (N = 36), inter-observer reliability was moderate (κ = 0.60) and substantial (κ = 0.72) across the two rounds, with no statistically significant difference (p = 0.250) (Table 5).

**Table 5:** Inter-observer reliability by classification types.

Category	N (cases)	Round	Overall Agreement (%)	Fleiss' Kappa	95 % CI	Z-value	p-value	4/4 Agreement (%)	3/4 Agreement (%)
Entire Classification	133	1	84.34	0.77	0.70–0.83	−1.84	0.066	72.9	95.5
		2	89.72	0.85	0.79–0.90			83.5	95.5
Diaphyseal Nonunions (All)	97	1	87.80	0.82	0.75–0.88	−1.84	0.066	76.3	97.9
		2	93.13	0.90	0.84–0.95			83.5	99.0
Segment Analysis – Femur	68	1 + 2	96.32	0.94	0.90–0.99	0.707	0.480	92.6	100
Segment Analysis – Tibia	48	1 + 2	93.75	0.91	0.84–0.98			87.5	100
Periarticular Nonunions	36	1	73.61	0.60	0.46–0.75	−1.15	0.250	52.8	88.9
		2	81.02	0.72	0.57–0.86			69.4	83.3

Groups.

For diaphyseal type A nonunions, only moderate agreement was observed in both rounds of assessment (k = 0.46 and 0.40 respectively). This may reflect the inherent complexity or subjectivity in evaluating type A nonunions.

For diaphyseal types B and C inter-observer reliability demonstrated moderate (k = 0.49) and substantial (k = 0.65) agreement after 1 and 2 rounds. The difference between rounds was not statistically significant (p = 0.077).

A similar trend was observed, with agreement improving from moderate (κ = 0.49) to substantial (k = 0.62) between the first and second rounds, although the difference was not statistically significant (p = 0.230) (Table 6).

**Table 6:** Inter-observer reliability by groups and subgroups.

Segment	N (cases)	Round	Overall Agreement (%)	Fleiss' Kappa	95 % CI	Z-value	p-value
*Groups*							
Diaphyseal (Type A)	36	1	63.98	0.46	0.29–0.63	0.55	0.580
		2	60.10	0.40	0.27–0.53		
Diaphyseal (Types B, C)	61	1	65.76	0.49	0.36–0.61	−1.77	0.077
		2	76.97	0.65	0.53–0.78		
Periarticular	36	1	66.20	0.49	0.34–0.65	−1.20	0.230
		2	74.54	0.62	0.47–0.76		
*Subgroups*							
Diaphyseal (Types A, B, C)	97	1	92.76	0.89	0.84–0.95	−1.38	0.168
		2	95.70	0.94	0.89–0.98		
Periarticular	36	1	94.44	0.89	0.78–0.99	N/A	N/A
		2	100	1.00	1.00–1.00		

Subgroups.

For diaphyseal nonunions, inter-observer agreement was consistently high in both assessment rounds (k = 0.89 and 0.94), demonstrating almost perfect agreement (p = 0.168).

For periarticular nonunions, inter-observer reliability was also almost perfect in both rounds (k = 0.89 and 1.00). The p-value comparison for the periarticular nonunion subgroup between Rounds 1 and 2 was not performed, as 100 % agreement among raters was achieved in Round 2, and no confidence interval was available. This precludes the use of a Z-test, which requires a non-zero standard error for valid computation (Table 6).

### 3.3 Intra-observer reliability

Intra-observer reliability for the classification by type was assessed for all four raters based on repeated evaluations of 133 cases after a two-week washout period. The results demonstrated substantial to almost perfect agreement across all observers.

Types.

For types of nonunions, Cohen's Kappa values ranged from 0.79 to 0.93, with overall agreement between 84.2 % and 96.2 %, indicating substantial to almost perfect agreement.

Groups.

Across all raters, intra-observer reliability for group classification ranged from moderate to substantial, with Cohen's kappa values of 0.55–0.74 and overall agreement of 74.0–82.4 %. These findings indicate that, although the classification system is generally stable, group-level differentiation remains more variable and may benefit from further refinement of clinical and radiographic criteria, particularly in borderline or complex cases.

Subgroups.

Intra-observer agreement for subgroup classification was consistently almost perfect across all raters. For diaphyseal nonunions, Cohen's kappa ranged from 0.89 to 0.95, with overall agreement between 86.6 % and 96.9 %. For periarticular nonunions, reliability was similarly high, with kappa values from 0.87 to 1.00 and agreement levels between 93.8 % and 100 %.

No statistically significant differences were observed between any pairs of kappa values after Bonferroni correction. Table 7 summarizes intra-rater reliability for all four raters across types, groups, and subgroups.

**Table 7:** Intra-observer reliability summary by rater and classification level.

	Rater 1	Rater 2	Rater 3	Rater 4
*Types*				
N	133	133	133	133
Cohen's Kappa	0.82 (almost perfect)	0.80 (substantial)	0.93 (almost perfect)	0.79 (substantial)
95 % CI	0.74–0.90	0.71–0.89	0.87–0.99	0.70–0.88
Agreement (%)	86.5 %	86.5 %	96.2 %	84.2 %
*Groups*				
N	131	129	131	127
Cohen's Kappa	0.73 (substantial)	0.62 (substantial)	0.74 (substantial)	0.55 (moderate)
95 % CI	0.63–0.84	0.51–0.74	0.65–0.83	0.42–0.68
Agreement (%)	82.4 %	76.0 %	79.4 %	74.0 %
*Subgroups – Diaphyseal*				
N	95	93	96	91
Cohen's Kappa	0.94 (almost perfect)	0.95 (almost perfect)	0.94 (almost perfect)	0.89 (almost perfect)
95 % CI	0.89–0.99	0.90–1.00	0.88–1.00	0.81–0.97
Agreement (%)	94.7 %	96.8 %	96.9 %	86.6 %
*Subgroups – Periarticular*				
N	34	32	36	33
Cohen's Kappa	0.93 (almost perfect)	0.87 (almost perfect)	0.88 (almost perfect)	1.00 (almost perfect)
95 % CI	0.81–1.00	0.71–1.00	0.73–1.00	1.00–1.00
Agreement (%)	97.1 %	93.8 %	94.4 %	100.0 %

### 3.4 Stage 3 – classification optimization

Implementation of the refined classification criteria resulted in a significant improvement in inter-rater reliability across all groups, as demonstrated by increased kappa values and statistically significant Z-test results. Results are summarized in Table 8.

**Table 8:** Inter-observer reliability for group classification before and after the introduction of refined criteria.

Group	Stage	N (cases)	Overall Agreement (%)	Fleiss' Kappa	95 % CI	Z-value	p-value
Diaphyseal (Type A)	2 (rounds 1 + 2)	72	61.57	0.42	0.32–0.53	4.58	<0.001
	3	30	87.78	0.82	0.68–0.95		
Diaphyseal (Types B and C)	2 (rounds 1 + 2)	122	71.39	0.57	0.49–0.65	2.67	0.0075
	3	30	86.11	0.79	0.65–0.93		
Periarticular	2 (rounds 1 + 2)	72	70.37	0.56	0.45–0.66	2.69	0.007
	3	30	86.67	0.80	0.66–0.94		

## 4 Discussion

Successful treatment of fracture nonunions requires a classification system that reliably reflects anatomical, biomechanical, and biological characteristics. Existing systems lack adequate reliability and universality for routine clinical or research use.^2^^,^^10^^,^^18^^,^^19^ The Universal Long Bone Nonunion Classification (ULBNC) follows the AO/OTA logic, linking anatomy with morphology to create a structured framework for clinical decision-making.^2^^,^^9^^,^^12^

A key innovation of ULBNC is incorporating pathological mobility (PM) into biological assessment. Ilizarov proposed a 7° threshold to distinguish “stiff” from “mobile” nonunions.^6^ PM reflects mechanical instability and soft-tissue vitality, forming a continuum from hypertrophic (stiff) to oligotrophic (lax) nonunions. ULBNC therefore classifies vital nonunions as hypertrophic, normotrophic, or oligotrophic. Although synovial pseudarthrosis may radiographically resemble a hypertrophic nonunion, its capsule-like structure and marked laxity make it biologically equivalent to oligotrophic.

For diaphyseal nonunions, the Weber–Cech system relies solely on radiographs and excludes PM — an essential criterion emphasized by Ilizarov and Paley.^4^^,^^6^^,^^7^ By integrating radiographic and clinical factors, ULBNC demonstrated excellent inter- and intra-rater reliability in our study, underscoring the value of incorporating PM.

**Fig. 8:**
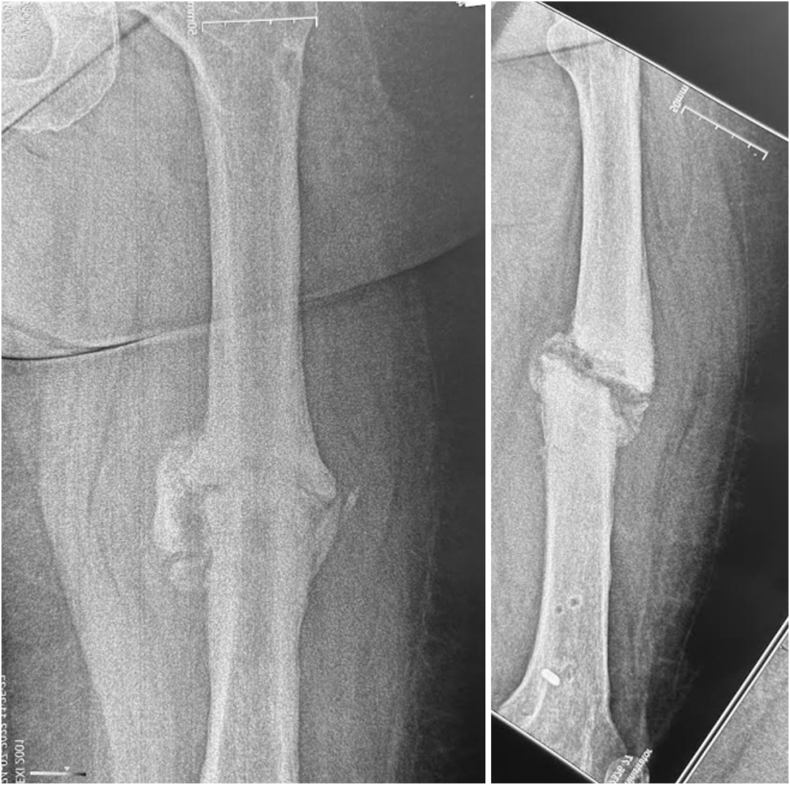
**Standardization of the definition of acceptable alignment.** A 56-year-old male with hypertrophic diaphyseal femur nonunion (ULBNC Type 32 A) and malalignment – angulation: 2°, shortening: 10 mm, translation: 5 mm. After Stage 2 validation, 2 out of 4 raters considered the alignment acceptable (A1), and 2 out of 4 considered it unacceptable (A2). After applying refinement criteria, it was clarified that all reference lines and angles were within normal limits, and a 10 mm shortening is acceptable. As a result, all four raters classified the case as A1.

**Fig. 9:**
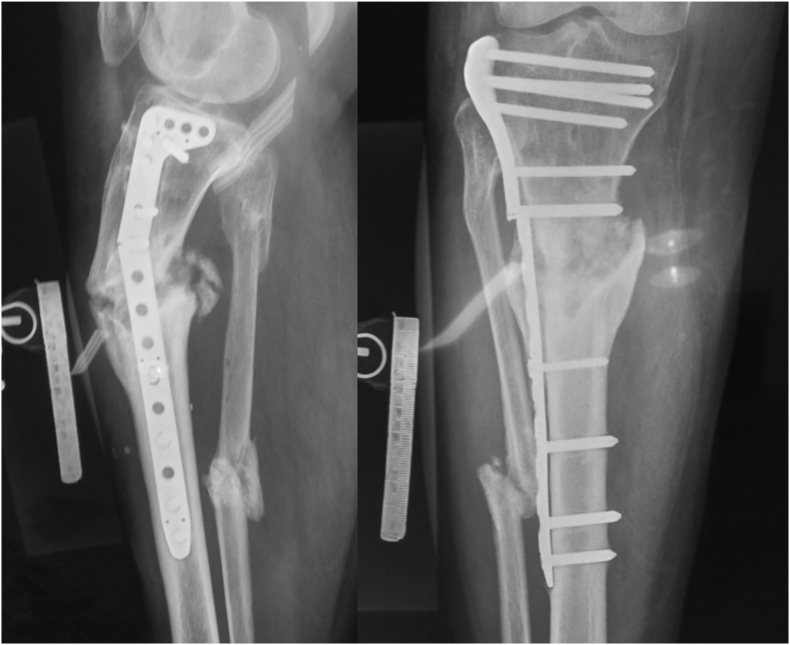
**Deformity correction strategy.** A 30-year-old female with hypertrophic diaphyseal tibial nonunion (ULBNC Type 4T2A) and malalignment – angulation: 23°, shortening: 15 mm, translation: 10 mm. After Stage 2 validation, 2 out of 4 raters classified it as a deformity that may be corrected acutely (A2), and 2 out of 4 as a deformity that should be corrected gradually (A3). After applying refinement criteria, all four raters accepted the borderline limit of 20° for acute deformity correction and classified the case as A3.

Group-level classification—based on fixation stability, alignment, and the planned correction strategy—proved clinically meaningful, though initial inter-observer agreement was only moderate. Lower agreement after Stage 2 stemmed from inconsistent definitions of acceptable alignment, differing criteria for acute versus gradual correction, and challenges in evaluating instability in periarticular cases. After refining and standardizing these criteria, reliability improved markedly. Examples of previously discordant cases and their resolution are shown in Fig. 8, Fig. 9.

Periarticular nonunions present unique challenges due to their proximity to joint surfaces. CT was essential for accurate assessment, and reliability for periarticular types, groups, and subgroups was high.

Subgroup classification based on fixation type showed the highest agreement, supporting its usefulness for clinical communication and research standardization.

It is important to emphasize, that atrophic and septic “nonunions” are not included in ULBNC as they represent a fundamentally different clinical problem and should be managed as segmental bone defects rather than nonunions.^2^^,^^4^ The management of nonunions is grounded in the “diamond concept,” which highlights the need to optimize both the mechanical and biological environments to achieve bone union.^20^ Cancellous autografting remains the “gold standard” for enhancing the biological component. However, “atrophic” and “infected” nonunions pose a significantly greater challenge, as the combination of bone grafting and stable fixation is insufficient in the presence of nonviable bone.

In such cases, treatment requires complete resection of all necrotic or infected bone, creating a segmental bone defect, regardless of whether the initial radiograph shows apparent contact between necrotic bone ends or a sequestrum. Reconstruction then follows established defect-management principles — either distraction osteogenesis (bone transport or shortening and re-lengthening) or direct defect reconstruction (Masquelet technique, vascularized or non-vascularized fibular grafting, etc.), selected according to defect size, anatomical location, and soft-tissue status. Clear communication is essential for appropriate clinical decision-making and to prevent medicolegal misunderstandings. The Universal Long Bone Defect Classification (ULBDC) therefore provides the appropriate framework for categorizing and managing these cases.^15^

## 5 Conclusion

The ULBNC demonstrates high inter- and intra-observer reliability and provides a clinically relevant, standardized framework for assessing nonunions. By incorporating anatomical location and key clinical parameters—pathological mobility, fixation stability, and alignment—it supports treatment planning and consistent comparison of outcomes. Its main limitation is relative complexity, which may restrict routine use to surgeons specializing in nonunion treatment.

## Ethical approval

Ethical approval was obtained from the Ethics Committee of the Medical Faculty of the Medical University - Sofia prior to the commencement of the study (Reference number: 2209-12/24).

## Patient/guardian consent

Due to the retrospective design of the study and the use of anonymized radiographic images, individual patient or guardian consent was not required. No identifiable personal data are included in this manuscript.

## Author contributions

**•**Conceptualization: [Anton A. Semenistyy], [Leonid N. Solomin]

**•**Methodology and Investigation: [Anton A. Semenistyy],], [Leonid N. Solomin]

**•**Data Curation and Formal Analysis: [Artem V. Komarov], [Roman Y. Mitsikov], [Borislav G. Tasev], [Andrey N. Mironov]

**•**Writing – Original Draft: [Anton A. Semenistyy]

**•**Writing – Review & Editing: All authors.

**•**Supervision: [Leonid N. Solomin]

All authors approved the final version of the manuscript and agree to be accountable for all aspects of the work.

## Funding statement

This research did not receive any specific grant from funding agencies in the public, commercial, or not-for-profit sectors.

## Declaration of interest

None.
